# Transition of Nano-Architectures Through Self-Assembly of Lipidated β^3^-Tripeptide Foldamers

**DOI:** 10.3389/fchem.2020.00217

**Published:** 2020-03-31

**Authors:** Nathan Habila, Ketav Kulkarni, Tzong-Hsien Lee, Zahraa S. Al-Garawi, Louise C. Serpell, Marie-Isabel Aguilar, Mark P. Del Borgo

**Affiliations:** ^1^Department of Biochemistry and Molecular Biology, Monash University, Clayton, VIC, Australia; ^2^School of Life Sciences, University of Sussex, Brighton, United Kingdom; ^3^Chemistry Department, Mustansiriyah University, Baghdad, Iraq; ^4^Department of Pharmacology, Monash University, Clayton, VIC, Australia

**Keywords:** peptide materials, self-assembly, foldamers, nanoindentation, nanofibers, nanobelts

## Abstract

β^3^-peptides consisting exclusively of β^3^-amino acids adopt a variety of non-natural helical structures and can self-assemble into well-defined hierarchical structures by axial head-to-tail self-assembly resulting in fibrous materials of varying sizes and shapes. To allow control of fiber morphology, a lipid moiety was introduced within a tri-β^3^-peptide sequence at each of the three amino acid positions and the N-terminus to gain finer control over the lateral assembly of fibers. Depending on the position of the lipid, the self-assembled structures formed either twisted ribbon-like fibers or distinctive multilaminar nanobelts. The nanobelt structures were comprised of multiple layers of peptide fibrils as revealed by puncturing the surface of the nanobelts with an AFM probe. This stacking phenomenon was completely inhibited through changes in pH, indicating that the layer stacking was mediated by electrostatic interactions. Thus, the present study is the first to show controlled self-assembly of these fibrous structures, which is governed by the location of the acyl chain in combination with the 3-point H-bonding motif. Overall, the results demonstrate that the nanostructures formed by the β^3^-tripeptide foldamers can be tuned via sequential lipidation of N-acetyl β^3^-tripeptides which control the lateral interactions between peptide fibrils and provide defined structures with a greater homogeneous population.

## Introduction

**Figure 1:**
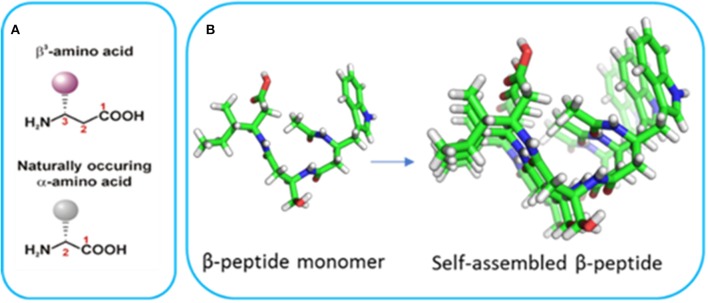
**(A)** The structure of α- and β-amino acids and **(B)** the crystal structure of a β^3^-tripeptide and the alignment of the side chains in a self-assembled system (reproduced with permission from Del Borgo et al., 2013).

Bottom-up nanofabrication of materials represents a powerful approach to generate tailored devices by taking advantage of the supramolecular self-assembly of molecules to form well-defined structures. Peptide-based self-assembly is the spontaneous formation of stable hierarchical structures via a combination of molecular interactions between the component peptides including hydrogen bonding, hydrophobic interactions, electrostatic interactions, π-π stacking, and van der Waals forces. The self-assembly of α-peptides has led to a wide range of peptide-based materials with a particular emphasis in tissue engineering applications (Cheng et al., 2013; Smith et al., 2016; Lee et al., 2017; Rodriguez et al., 2018). However, these materials are not suitable for many longer-term applications due to proteolytic degradation. Furthermore, materials derived from α-peptides typically require specific sequences to initiate self-assembly and the introduction of functionality can hinder self-assembly. In contrast, β-peptides are polyamides comprised exclusively of β-amino acids (Figure 1; Cheng et al., 2001; Gopalan et al., 2015) which differ from α-amino acids due to the presence of an extra methylene between the carboxyl group and α-carbon atom (Seebach and Gardiner, 2008; Torres et al., 2010). The side chains can be positioned at either the α- or β-carbon, resulting in either β^2^ or β^3^-amino acids. β^3^-Peptides have been shown to adopt well-defined helical structures stabilized by hydrogen bonding interactions (Appella et al., 1997; Seebach et al., 2006; Gopalan et al., 2012). Peptides comprised solely of acyclic β^3^-amino acids predominantly adopt a 14-helical conformation (Raguse et al., 2001; Seebach et al., 2006; Bergman et al., 2009). The 14-helix is characterized by ~3 residues per turn which results in the alignment of the side chains along the face of the helix (Figure 1B). These β^3^-peptide foldamers give rise to the assembly of an array of structures ranging from bundles with catalytic activity (Molski et al., 2010; Melicher et al., 2013; Wang et al., 2014; Wang and Schepartz, 2016) to irregular structures with paramagnetic properties (Kwon et al., 2015). Even more significantly in the context of material nanofabrication, we have shown that when N-acetylated, β^3^-peptides spontaneously self-assemble to form fibrous architectures with a large range of irregular architectures and sizes (Del Borgo et al., 2013). For example, a β^3^-tripeptide with the sequence β^3^Leu-β^3^Ile-β^3^Ala (LIA), was recently reported to self-assemble into a 14-helical coiled coil to form diverse morphologies in different solvents (Christofferson et al., 2018). Similarly, N-acetyl β^3^-peptides with the same β^3^-amino acid composition but different sequences also formed fibrous structures demonstrating sequence independent self-assembly that is unique to this class of peptides (Seoudi et al., 2015). The insertion of organic molecules between two β^3^-tripeptides did not prevent axial self-assembly, demonstrating how the tripeptides act as sticky ends due to the strength of the head-to-tail H-bonding motif (Del Borgo et al., 2018). The resulting fibrous scaffolds were also used as a biomaterial to successfully grow and maintain primary neuronal cultures. However, the considerable range in size of the resultant fibers from nano to centimeter limits the application of N-acetyl β^3^-peptide-based materials. Therefore, in the context of material nanofabrication, given that the control of self-assembly to yield defined fiber architectures is central to future applications, there is a need to develop new strategies for finer control of these materials. The unique 14-helical self-assembly that results in lateral alignment of side chains with a high degree of symmetry along the periphery of the β^3^-peptide nanofibrils (Christofferson et al., 2018) (Figure 1) now provides the opportunity to functionalize the peptide laterally without perturbing the axial self-assembly.

We have previously prepared a series of N-acetyl β^3^-tripeptides with the cell adhesion motifs, RGD and IKVAV, attached laterally via the amino acid side chain (Luder et al., 2016). We also demonstrated that the hydrophobic IKVAV-functionalized β^3^-peptide self-assembled axially to form long but thin fibers where the lateral assembly was restricted to just two intertwined nanofibrils (Luder et al., 2016). Thus, while the axial head-to-tail self-assembly is the dominant process leading to fibrils, lateral assembly between growing fibrils can be modulated by varying the side chain structure or solvent polarity to achieve control of fiber morphology. This suggested that attachment of a long, hydrophobic sequence to the side chain of a β^3^-amino acid residue within the peptide could potentially control the β^3^-peptide lateral self-assembly and ultimately fiber morphology.

Several studies have demonstrated that incorporation of an alkyl chain within a self-assembling α-peptide is a method to control self-assembly to form cylindrical nanofibers with uniform diameter (Hartgerink et al., 2002; Cui et al., 2010; Pashuck et al., 2010; Lee et al., 2011; Ortony et al., 2014). Here we describe a series of sequentially-lipidated β^3^-peptide foldamers (listed in Table 1) to give controlled self-assembled structures. We investigated the effect of lipid chain length as well as the location of the lipid within the peptide sequence. Moreover, the internal structure of resultant nanobelts was investigated in solution by high resolution AFM to reveal a unique multilaminar architecture driven by electrostatic forces.

**Table 1:** List of all β^3^-tripeptides designed and synthesized in this study [where Lau, Myr and Pal refer to lauric (C12), myristic (C14), and palmitic (C16) acid, respectively], and a summary of the nanoarchitectures formed (Az refers to β-homoazidoalanine).

**Peptide^**#**^**	**Sequence**	**Lipid location**	**Acyl chain length**	**Morphology**	**Ave. fiber height** **(nm)**
1	Ac-AzKA-OH	N/A	N/A	Dendritic	N/A
2	H_2_N-Az(Myr)KA-OH	AA1	14	N/A	N/A
3	Lau-KAK-OH	N-terminus	12	Twisted fibril	5.4
4	Myr-KAK-OH	N-terminus	14	Twisted fibril	6.4
5	Pal-KAK-OH	N-terminus	16	Twisted fibril	8.4
6	Ac-Az(Lau)KA-OH	AA1	12	Twisted fibril	5.9
7	Ac-Az(Myr)KA-OH	AA1	14	Twisted fibril	6.2
8	Ac-Az(Pal)KA-OH	AA1	16	Twisted fibril	8.4
9	Ac-AAz(Lau)K-OH	AA2	12	Flat Nanobelt	18.5
10	Ac-AAz(Myr)K-OH	AA2	14	Flat Nanobelt	22.4
11	Ac-AAz(Pal)K-OH	AA2	16	Flat Nanobelt	37.8
12	Ac-KAAz(Lau)-OH	AA3	12	Nanobelt	23.9
13	Ac-KAAz(Myr)-OH	AA3	14	Nanobelt	32.6
14	Ac-KAAz(Pal)-OH	AA3	16	Nanobelt	84.6
15	Pal-RAR-NH2	N-terminus	16	Fibrillar	15.1
16	Ac-Az(Pal)RR-NH2	AA1	16	Fibrillar	4.8
17	Ac-RAz(Pal)R-NH2	AA2	16	Fibrillar	21.3
18	Ac-RRAz(Pal)-NH2	AA3	16	Fibrillar	8.5

## Experimental

### Peptide Synthesis and Purification

Peptides 1–14 were synthesized on a 0.1 mmol scale using standard Fmoc chemistry on Wang resin (0.1 mmol/g loading). The resin was swollen in DMF (4 mL) and then soaked in Fmoc-protected β amino acid (2.1 eq. to resin loading), dissolved in DMF (4 mL) along with HBTU (2 eq. to resin loading), HOBt (2 eq. to resin loading), DMAP (10 mol%) and DIPEA (3 eq. to resin loading), overnight with gentle agitation. The resin was thoroughly washed with DMF (3 × 4 mL) and the Fmoc protecting group on the amino acid was removed by soaking the resin twice in 20% piperidine, with 0.1 M HOBt, in DMF (4 mL) for 15 min each. The resin was washed with DMF (3 × 5 mL), soaked in Fmoc-protected amino acid (2.1 eq. to resin loading), dissolved in DMF (4 mL) along with HBTU (2 eq. to resin loading), HOBt (2 eq. to resin loading) and DIPEA (3 eq. to resin loading), for 2 h. β Peptide elongation cycle was then repeated until the sequence was complete. After removing the terminal Fmoc protecting group on the peptide, the resin was treated with a solution of 10% v/v acetic anhydride and 2.5% v/v DIPEA in DMF (4 mL) for 30 min to afford an acetyl-capped N terminus. The resin was washed with DMF (2 × 4 mL), CH2Cl2 (2 × 4 mL), Et2O (2 × 4 mL), air dried for 10 min, and transferred to a 15 mL vial for further manipulation.

To facilitate attachment of the desired aliphatic chain, reduction of the azido-alanine residue on the β3-peptide was effected on solid support. The resin (0.1 mmol) was swollen in THF (1 mL) and then soaked in a solution of PPh3 (4 eq. to resin loading) in THF (2.5 mL) and H2O (50 μL), sealed in a capped vial and heated to 65°C, for 4 h. The resin was filtered through a sintered glass funnel and washed with THF (2 × 4 mL) and DMF (2 × 4 mL). The resin was then soaked in either lauric or myristic acid (3.1 eq. to resin loading), dissolved in DMF (4 mL) along with HBTU (3 eq. to resin loading), HOBt (3 eq. to resin loading), and DIPEA (4.5 eq. to resin loading), for 2 h. To couple palmitic acid, the resin was soaked in palmitic acid (2.1 eq. to resin loading), HBTU (2 eq. to resin loading), HOBt (2 eq. to resin loading), and DIPEA (6 eq. to resin loading), twice for 2 h. The resin was subsequently washed with DMF (2 × 4 mL), CH2Cl2 (2 × 4 mL), Et2O (2 × 4 mL), air dried for 10 min, and transferred to a 15 mL vial for cleavage.

Cleavage was performed on the resin (0.1 mmol), by treating the resin with a cleavage solution (10 mL) comprising of H2O (2.5% v/v), triisopropylsilane (2.5% v/v) in CF3COOH, for 3 h. CF3COOH was then evaporated under a stream of N2 and the peptide was precipitated by addition of Et2O (50 mL). The precipitate was filtered and redissolved in 50% aqueous CH3CN for lyophilisation. The peptide was redissolved in 60% aqueous CH3CN (5 mL) and purified by injecting the sample onto a reverse-phase preparative column, eluted over a 60 min gradient from 10 to 70% solvent B (solvent A: 0.1% TFA/H_2_O; solvent B: 0.1% TFA/CH_3_CN), with a flow rate of 6 mL/min. The fractions were collected and analyzed for purity by injecting the samples onto a reversed phase analytical column, eluted over a 45 min gradient from 0 to 75% solvent B (solvent A: 0.1% TFA/H_2_O; solvent B: 0.1% TFA/CH_3_CN), with a flow rate of 1 mL/min. Pure fractions were pooled. HPLC and MS data obtained are detailed in Table S1 and Figure S1.

### AFM

All samples were dissolved in water to a final concentration of 0.25 mg/mL and incubated for at least 1 h. Thereafter, 2 μL of samples were placed on a freshly cleaved 12 mm mica and air dried at room temperature. Structural analysis was performed using AFM in air with Nano-Scope® IV equipped with a MultiMode™ head (Veeco Instrument Inc. New York, USA and a Bruker AFM multi-mode VIII (Bruker Corporation Massachusetts, USA) powered by Peak Force® Tapping mode with ScanAsyst. Images were obtained using a “J-scanner” or “E-scanner.” The probe used was a mikromasch cantilever (NSC-15 “B” silicon cantilevers) with a force constant of 40 N/m. Topo-graphic, phase and amplitude images were captured simultaneously using a scan frequency of 1 Hz. The captured images were processed and height values measured using Gwyddion 2.45 software. One-hundred different fiber heights were measured by extracting the profiles before exporting to Microsoft Excel in order to produce normalized height profile graphs. Finally, statistical analysis was done using one-way ANOVA and Tukey's multiple comparison test.

### pH Switching of Morphology by AFM

All peptides were dissolved in phosphate buffer pH 4, 7 and aqueous 0.1 M NaOH pH 13 to a final concentration of 0.25 mg/mL and incubated for 24 h. Thereafter, 2 μL of incubated samples were placed on a freshly cleaved 15 mm mica surface. Structural analysis was carried out using AFM in fluid whereby the topographic, phase and amplitude images were captured. In addition, the height values of the nanofibers were determined for all peptides in each pH condition as previously described above.

### AFM Nanoindentation

Nanoindentation was investigated using an OLTESPA-R3 0.01–0.02 ohm-cm silicon probe (Bruker, Billerica, MA, USA) with a spring constant of 2 N/m, resonance frequency of 70 kHz and cantilever tip radius of 7 nm. Peptide 11 was dissolved in distilled water and imaged using a fluid cell. Nanoindentation was performed by precisely punching the AFM tip into the surface of a nanobelt by first maneuvering the AFM tip to the center of the nanobelt and then executing a force ramp with 250 nm trigger threshold. The topographic, phase and amplitude images were captured simultaneously, and processed using Gwyddion 2.45 software.

### Quantitative Nanomechanical Mapping

The peak force quantitative nanomechanical mapping (PF-QNM) was performed in a fluid cell containing water using the OLTESPA-R3 probe on a MultiMode 8 AFM. To ascertain deflection sensitivity, the AFM tip was first calibrated using a clean sapphire surface and secondly by a polystyrene surface standard. Thereafter, surface topology and Young's modulus mapping were performed for peptides 3, 9, and 10 at a scan rate of 1 Hz. A line cross section was made on 5 individual fibers and the raw mean modulus values were used. All images were captured simultaneously with typical scan sizes of ≤ 10 μm. Images were captured and analyzed using the software Nanoscope analyser and Gwyddion 2.45 software.

### Transmission Electron Microscopy

Structural analysis was carried out by TEM using 2 μL peptide sample solution deposited onto a carbon-coated copper grid. The excess solution was gently blotted with filter paper and left to dry for 30 min under ambient conditions. Microscopy was done on a Hitachi H-7500 TEM (80 keV) and a FEI Tecnai G2 Spirit TEM (Oregon, USA) operated at 100 and 120 Kev. The captured images were processed and analyzed using ImageJ software.

### Circular Dichroism Analysis

Peptides were dissolved in milli-Q distilled water and incubated at 37°C overnight. Eleven formed a precipitate so was centrifuged at 13,000X rpm for 3 min to reduce scatter and remove particles. Far UV CD spectra were collected using a 1 mm path length quartz cuvette and JASCO J-715 spectropolarimeter using 4 s response time, 50 nm/min scan rate, 1 nm bandwidth and a data pitch of 0.1 mm. Each scan was collected from 180 to 250 nm using three acquisitions and a peltier device maintained the temperature at 20°C. Raw CD spectra were converted from millidegrees to molar ellipicity per residue (MER), except for **11** which is in millidegrees due to the unknown concentration after centrifugation.

## Results and Discussion

### Lipidation of Tri-β^3^-Peptide Monomers Leads to Fibers of Uniform Diameter

The overall goal of this study was to control the lateral self-assembly of β^3^-peptides to form homogeneous and well-defined architectures. We have previously reported a lipidated N-acetyl β^3^-tripeptide **7** that contains a tetradecyl (C14) chain at amino acid position 1, which formed a biocompatible hydrogel able to support the growth of cells (Motamed et al., 2016), and was further functionalized to incorporate bioactive peptide ligands within the β^3^-peptide scaffold (Kulkarni et al., 2016). In order to investigate the structure and hierarchical assembly of this class of β^3^-peptides, we utilized lipids of varying length within the tripeptide sequence, listed in Table 1, to determine the effect of alkyl chain length and position on directing self-assembly and analyzed these structures by AFM and TEM, the results of which are also summarized in Table 1.

**Figure 2:**
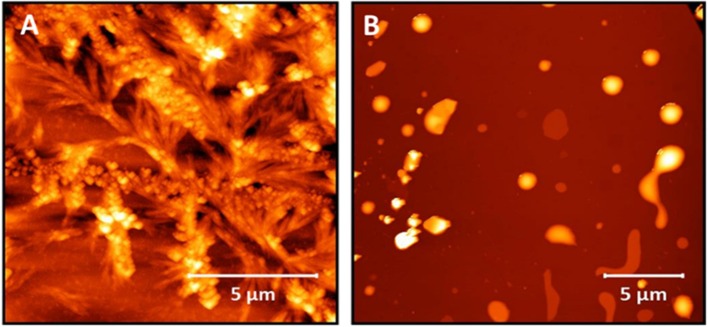
AFM images of **(A)** peptide **1**, which does not contain an acyl chain showing the uncontrolled nature of the self-assembly and **(B)** peptide **2** showing no fiber formation.

We have previously shown non-lipidated N-acetyl β^3^-peptides self-assemble into largely dendritic structures with very little control over the dimensions or topology of the structure (Del Borgo et al., 2013). As a control for the present study, the non-lipidated N-acetyl β^3^-tripeptide **1** was synthesized, and the results in Figure 2A confirm the formation of dendritic structures by the non-lipidated peptide template. A second control lipidated peptide **2**, which was not acetylated at the N-terminus was also synthesized and the absence of fibers evident by AFM analysis shown in Figure 2B, further demonstrates the crucial contribution of the N-terminal amide to the self-assembly.

**Figure 3:**
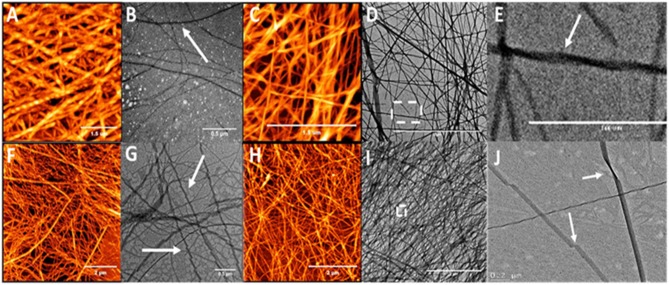
AFM and TEM analysis of the twisted fibrous structures formed by the self-assembly of peptide **4 (A,B)**, **5 (C–E)**, **7 (F,G)**, and **8 (H–J)** which are lipidated at the N-terminus or position 1 (N-terminal region); arrows indicate twisting.

In order to study the effect of alkyl chain length and the position of the alkyl chain on self-assembly, a series of β^3^-peptides (**3–14)** was synthesized in which the alkyl chain length was either 12, 14, or 16 carbons in length, and placed at the N-terminus or amino acid position 1, 2 or 3 (Table 1). AFM and TEM images for the C14- and C16-coupled peptides are shown in Figure 3 while the AFM analysis of peptides **3**, **6**, **9**, and **12** containing a 12-carbon chain are shown in Figure S2. Overall, three main conclusions can be drawn from the data shown. Firstly, the results show that the presence of either a 12, 14, or 16 carbon chain resulted in fibers of reasonably uniform diameter rather than the dendritic and macroscopic fibers formed from the self-assembly of non-lipidated N-acetyl β^3^-peptides (Del Borgo et al., 2013). Secondly, the results revealed that incorporation of a lipid chain in the peptide monomer at any position within the sequence did not disrupt self-assembly. Thirdly, AFM and TEM analysis of all lipidated N-acetyl β^3^-peptides revealed that the length of the lipid chain at each sequence position had no observable effect on the fiber diameter, nor any effect on the overall morphology of the self-assembled fiber architecture. Thus, the controlled assembly of these fibers resulted in a more homogenous self-assembly that inhibited the formation of large, undesirable macroscopic fibers.

Closer analysis of the AFM images for peptides **3–14** reveals additional structural insight into the packing of the fibers. Peptides lipidated in the N-terminal region with either a C14 or C16 chain at either the N-terminus (Peptides **3** and **4**) or on the side chain of the first β^3^-amino acid residue (Peptides **6** and **7**), formed an intertwined nanofibrous mesh with surface topography of these nanofibers showing a distinct periodicity (Figure 3). Higher resolution imaging revealed the periodicity to be twists along the fibril surface (e.g., Figure 3A) and the fibers measured between 6 and 7 nm in height (Figure S3). The effect of alkyl chains on the peptide self-assembly in this study is also consistent with previous reports in which alkyl chains of at least 6 carbons were required to control the self-assembly of lipidated α-peptides, also known as peptide amphiphiles (Gore et al., 2001; Hartgerink et al., 2002; Malkar et al., 2003; Palmer and Stupp, 2008; Lee et al., 2011; Miravet et al., 2013; Dube et al., 2014; Korevaar et al., 2014; Ortony et al., 2014; Hamley, 2015).

In most α-peptide amphiphiles the hydrophobic alkyl chain is typically introduced by coupling palmitic acid (C16) at the N-terminal amine of a short peptide (Stendahl et al., 2006; Missirlis et al., 2011; Hamley et al., 2013; Miravet et al., 2013; Moyer et al., 2013; Stupp and Palmer, 2014). Other reports have also shown that the control of α-peptide amphiphile assembly was not only due to the incorporation of alkyl chains, but also due to variation of sequence length (Marullo et al., 2013), or by incorporating specific residues within the peptide sequence (Löwik et al., 2005; Nieuwland et al., 2013; Hamley et al., 2015). Our results demonstrate that the lipid chain can be coupled at any residue without loss of self-assembly, however, the location of the acyl chain allows a transition of the nanoarchitecture from fibers to belts in peptides with identical elemental composition. Therefore, the position of the acyl chain within the peptide sequence acts as a supramolecular switch.

**Figure 4:**
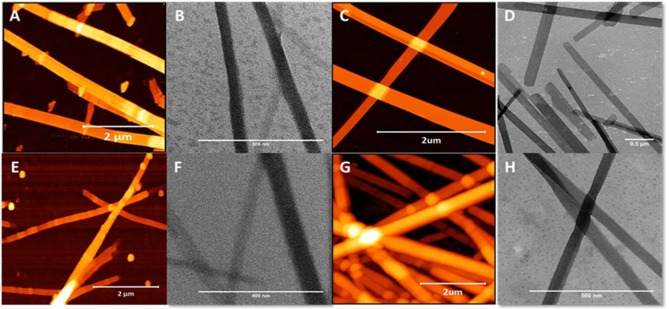
AFM and TEM analysis of the nanobelts formed by the self-assembly of peptide **10 (A,B)**, **11 (C,D)**, **13 (E,F)**, and **14 (G,H)**, which are lipidated at positions 2 or 3 (C-terminal region).

In stark contrast to β^3^-peptides lipidated at the N-terminal region, β^3^-peptides lipidated in the C-terminal region on the side chain of either the second or third β^3^-amino acid residues (peptides **3** and **4**) formed flat, long and straight nanobelts, without any intertwining (Figure 4). The TEM images obtained under the same conditions further confirmed the nanobelt morphology (Figure 4). Most importantly, the nanobelts assembled through lipidation of the C-terminal residue (residue 3) are distinct from those formed by lipidation of the second amino acid. Lipidation at residue 2 results in wider nanobelts that appear flat on the surface. In contrast, nanobelts formed by acylation of the C-terminal residue (residue 3) were larger in the z-axis.

Previous studies of self-assembled α-peptide amphiphiles have predominantly exploited lateral non-covalent interactions (Capito et al., 2008; Zhao et al., 2010; Lin et al., 2011; Dehsorkhi et al., 2013; Garifullin and Guler, 2015; Zhang et al., 2016). Specifically, the driving forces that govern self-assembly of α-peptide amphiphiles to yield cylindrical nanofibers arise from the combined effect of hydrophobic interactions of the alkyl chains, hydrogen bonding among the middle peptide segments and electrostatic interactions between the charged amino acids (Hartgerink et al., 2002; Velichko et al., 2008; Hamley, 2011; Cui et al., 2014; Da Silva et al., 2016). A number of studies have also proposed that the presence of hydrophobic alkyl chains on α-peptide amphiphiles that are screened in aqueous environments, result in a rod-like shape that eventually forms fibers with the hydrophilic peptide component presented on the surface while the hydrophobic alkyl chains pack in the core of the structure (Cui et al., 2010, 2014; Lin et al., 2011).

In contrast, the unique 14-helix which underpins the self-assembly motif of the N-acyl β^3^-peptides gives rise to a unique geometry where the side-chain of each β^3^-amino acid residue is aligned with the corresponding amino acid residue of the axially proximal β^3^-peptide monomer in the self-assembled nanorod (Figure 1B). CD spectra of peptides **7**, **10** and 11 are shown in Figure S4 and all three peptides exhibit a minima in the region between 210 and 215 nm. This spectral feature suggests the presence of secondary structure which in the case of β^3^-peptides is consistent with a 14-helix (Bergman et al., 2009; Gopalan et al., 2012).

As a consequence, for the peptides used in this study, it can be proposed that the lipid side chains are all positioned on the same face of the 14-helix which provides the potential for the lipid chains are likely to align and pack in a layered arrangement. the orientation of the lipid chain on the surface of a fibril influences the lateral interactions This alignment then allows for fine control of the supramolecular structures formed by either limiting or promoting the amount of non-covalent interactions that drive lateral self-assembly and results in greater uniformity in fiber morphologies than previously described β^3^-peptides (Del Borgo et al., 2013, 2018; Seoudi et al., 2015, 2016; Luder et al., 2016; Christofferson et al., 2018). The current results for the lipidated N-acetyl β^3^-tri-peptide foldamers therefore demonstrate a powerful design strategy to control fiber morphology through variation in the position of the alkyl chain.

### Internal Architecture of Nanobelts Using Nanoindentation

**Figure 5:**
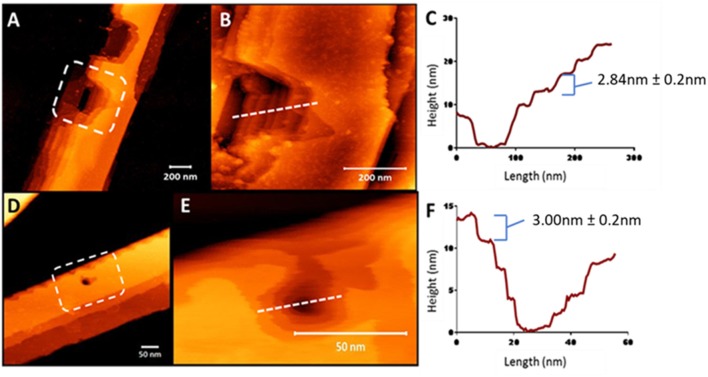
AFM nanoindentation on the surface of peptide nanobelts **10 (A)** and **11 (D)** in air revealing the internal multilaminar stacking nanoarchitecture and in a hydrated system (**B,E**, respectively). Height profiles of these indented nanobelts from **10 (C)** to **11 (F)** indicating 3 nm steps.

To understand the surface features of the nanobelts, topological profiles for peptides were extracted and graphically represented (Figures S5, S6), which revealed the surface of the nanobelts to be flat with some measuring over 100 nm in height and a width >250 nm. In order to investigate the fibril stacking within the large nanobelts, the tip of the AFM probe was used to punch a hole into the surface of the nanobelt to reveal the internal packing arrangement. Clear evidence of multilaminar stacking was evident with each layer measuring approximately 3 nm (Figure 5). A previous report of an α-peptide amphiphile with the sequence C16–VEVE-OH revealed long nanobelts that were formed after 2 days with heights between 10 and 20 nm 58 indicating the stacking of 2–3 layered structures. In comparison, peptides **10**, **11**, **13**, and **14** in this study spontaneously formed nanobelts that measured up to 150 nm in height, which corresponds to approximately 50 layered structures.

The dimensions of multilaminar stacking within the nanobelt structure strongly suggests the formation of peptide layers, which most likely interact via electrostatic interactions between the C-terminal carboxylate and the protonated ε-amine of the lysine sidechain. Interestingly, the force required to indent these nanobelts increased as the acyl chain length increased and AFM analysis using peak-force quantitative nanomechanical mapping (PF-QNM), demonstrated that increasing the lipid chain length directly correlated with an increase in fiber stiffness (Figure S7).

### Electrostatic Control of Fiber Morphology

**Figure 6:**
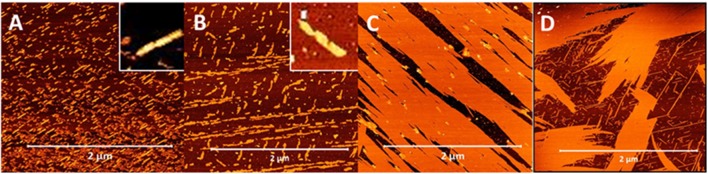
AFM images demonstrating the modulation of self-assembled nanoarchitecture of β^3^-peptides **5 (A)**, **8 (B)**, **11 (C)**, and **14 (D)** through changes in pH; insets in **(A,B)** highlight twisting morphology.

Given the vast differences in fiber morphology within the series of lipidated N-acetyl β^3^-peptides, the contribution of the lateral electrostatic interactions to the overall fiber morphology was further investigated using changes in pH. Peptides **5, 8, 11**, and **14** (C16 series) were incubated under acidic conditions (pH 4) and the resulting fibers were visualized using AFM as shown in Figure 6. Peptides **5** and **8** (lipidated at the N-terminus and residue 1, respectively), formed truncated fibrils at pH 4 (Figures 6A,B), in strong contrast to the interwoven fibrous mesh observed at pH 7 (Figure 3). The short fibrils at pH 4 were also twisted (inset Figures 6A,B), and similar to the twisted fibrous structures initially observed for these peptides (Figures 3E,G,J). This further demonstrates that electrostatic interactions result in a persistent twist within the fibrils even after truncation due to pH driven partial inhibition of the axial 3-point H-bonding.

The distinctive nanobelts arising from lipidation at the C-terminal region of the β^3^-tripeptide (peptides **11** and **14**) were also analyzed under acidic conditions and shown in Figures 6C,D. At neutral pH, these zwitterionic peptides formed multilaminar assemblies in the z-axis promoted by lateral electrostatic attraction between the peptide layers. However, the thick, tall nanobelts formed by peptides **11** and **14** at pH 7 were completely inhibited at pH 4. Under acidic conditions, the long-range electrostatic interactions, which are pH dependent, are inhibited, thereby resulting in the loss of stacking assembly in the z-axis. Consequently, these peptides only associate laterally to form a film-like monolayer on the surface (Figures 6C,D), as evident from the height measurements (~3 nm). These results are also consistent with the 3 nm steps found by AFM nanoindentation (Figure 5) of the larger nanobelt structures.

**Figure 7:**
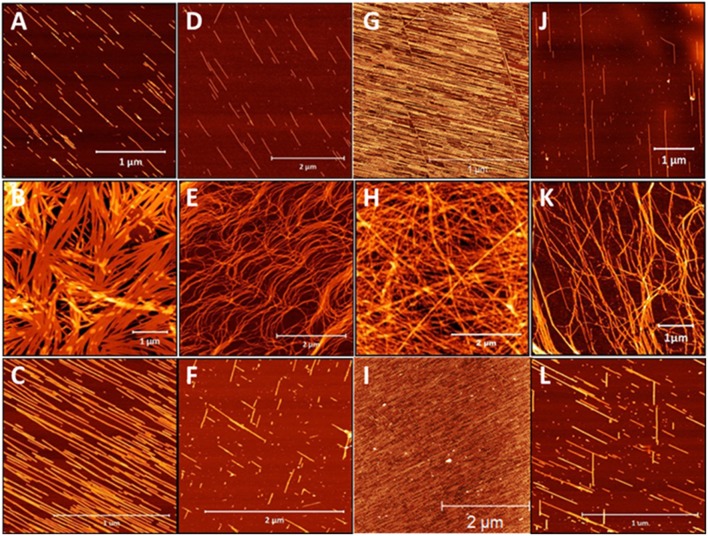
AFM images of the architectures derived from the self-assembly of β-peptides containing multiple positive charges **(A–C)** peptide **15**, **(D–F)** peptide **16**, **(G–I)** peptide **17**, and **(J–L)** peptide **18** demonstrating the influence of pH on these assemblies (top panel pH 4, middle panel pH 7, and bottom panel pH 13).

Given the striking influence of pH on the β^3^-peptide morphologies resulting from changes in lateral electrostatic interactions within the peptide assemblies, a series of peptides was designed that were positively charged under all pH conditions. Peptides were synthesized with a C-terminal amide and the sequence comprising only arginine residues and a palmitoyl chain that was positioned at the N-terminus or residues 1, 2, or 3 of the β^3^-tripeptide, to yield peptides **15–18** (Table 1). The nanoarchitectures formed under the same pH conditions were then assessed to determine the effect of inhibiting the lateral self-assembly of nanofibers on the patterned surfaces. All four arginine-containing β^3^-peptides **15–18** formed a fibrous mesh when dissolved in a pH 7 buffer (Figures 7B,E,H,K) which were similar to those observed for the N-terminal region lipidated *N*-acetyl β^3^-peptides (Figure 3). Interestingly, there was no evidence of any nanobelt formation at pH 4, 7, or 13 for any *N*-acetyl β^3^-peptides of this group. Most striking were the architectures formed by the peptides at pH 4 and 13 (Figures 7G–L). These fibrils, particularly those formed by the self-assembly of peptides **15** and **17** (Figures 7A,C,G,I), appeared directionally aligned with little, if any, crossover of fibers in the sample.

These conditions generally produced truncated rod-like nanofibers, without any noticeable twisting, presumably due to the suppression of axial H-bonding and via electrostatic repulsion. At neutral pH a nanofibrous mesh was produced regardless of the alkyl chain location suggesting that the C-terminal acid, and hence the lateral electrostatic interactions, are critical to the formation of nanobelts. In addition, the alignment of nanofibers observed for these β^3^-peptides at pH 4 and pH 13 was most likely due to the surface charge introduced within the peptide monomer by the addition of arginine residues (Figures 7A,C,D,F). This results in electrostatic repulsion due to the presence of a large number of positive charges along the surface of each nanofibril. The electrostatic repulsion is sufficient to inhibit any fiber crossover and is responsible for generating aligned fibers best exemplified by peptides **13** and **15** (Figures 7A,C,G,I).

Previous reports with α-peptide amphiphiles demonstrated the alignment of nanofibers using external triggers including dip-pen nanolithography (Jiang and Stupp, 2005), ultrasonication (Hung and Stupp, 2009) or a magnetic field (Reches and Gazit, 2006). The generation of regular, aligned structures simply through the design of positively charged β^3^-peptide monomers through control of the surface charge is an exciting development with applications in nanotechnology.

## Conclusions

We have described a series of lipidated β^3^-tripeptides that form regular fibrous structures of controlled dimensions. These fibrous assemblies can be tuned by altering the position of the lipid within the β^3^-peptide sequence to produce an interwoven mesh or ultra-wide multilaminar nanobelts. Fiber dimensions were quantitated using high resolution AFM, which has provided the first insight into the internal packing of these materials. The internal structure of these nanobelts were revealed to have regular 3 nm layers that were completely inhibited through changes in pH. Additional fiber diffraction and modeling is underway that will provide further insight into the assembly process. Ultimately, we believe these results will provide a promising strategy using a bottom-up approach to create tailored self-assembled materials with potential in a broad range of bio- and nanomedicine applications.

## Data Availability Statement

All datasets generated for this study are included in the article/Supplementary Material.

## Author Contributions

MD, T-HL, LS, KK, and M-IA conceptualized the ideas. NH, KK, and ZA-G performed the experiments. MD wrote the first draft of the manuscript. All authors provided revisions and analyzed data.

### Conflict of Interest

The authors declare that the research was conducted in the absence of any commercial or financial relationships that could be construed as a potential conflict of interest.

## Abbreviations

AFM: atomic force microscopy
TEM: transmission electron microscopy
CD: circular dichroism.
