# Supplementary material for: Transition of Nano-Architectures Through Self-Assembly of Lipidated β3-Tripeptide Foldamers
Source: Front Chem. 2020 Mar 31;8:217. doi: 10.3389/fchem.2020.00217 (PMC7136582; doi:10.3389/fchem.2020.00217)
Supplement: Supplementary file 1 [file Table_1.DOCX]

Supporting Information for;

Transition of Nano-Architectures Through Self-Assembly of

Lipidated β^3^-Tripeptide Foldamers

*Nathan Habila^1#^, Ketav Kulkarni^1#^, Tzong-Hsien Lee^1^, Zahraa S. Al-Garawi^3,4^, Louise C. Serpell^3^, Marie-Isabel Aguilar^1^* and Mark P. Del Borgo^1,2^**

^1^ Department of Biochemistry and Molecular Biology and ^2^ Department of Pharmacology, Monash University, Clayton, VIC, 3800, Australia

^3^ School of Life Sciences, University of Sussex, Falmer, East Sussex BN1 9QG, UK.

^4^ Chemistry Department, Mustansiriyah University, Baghdad, Iraq

**Table of Contents**

**Table S1:** MS data of all peptides ……………………………………………..………..………S2

Supporting Figures

**Figure S1**: HPLC of all peptides………………...………………...……….….………………….S3

**Figure S2**: AFM images of peptides 3,6,9 & 12.…………...………………….……..…..S4

**Figure S3**: AFM height profiles of peptides 4,5,7 & 8………….…………..……..…..S5

**Figure S4:** CD analysis of lipidated peptides 7, 10 and 11……………………………S6

**Figure S5**: AFM topological traces of peptides 10 & 11..………..…….…...…..…..S7

**Figure S6**: AFM topological traces of peptides 13 & 14……….………..………..…..S8

**Figure S7**: Nanobelt stiffness of peptides 5, 10 & 11…....……………………..….....S9

**Table 1**: List of MS data obtained for synthesised peptides

| Peptide # | Mass Calc. | Mass Obs. |
| --- | --- | --- |
| 1 | 413.3 | 413.1 |
| 2 | 555.3 | 555.4 |
| 3 | 569.5 | 569.3 |
| 4 | 597.5 | 597.3 |
| 5 | 625.5 | 625.4 |
| 6 | 569.4 | 569.3 |
| 7 | 596.3 | 596.5 |
| 8 | 625.5 | 625.5 |
| 9 | 569.4 | 569.4 |
| 10 | 596.5 | 596.4 |
| 11 | 625.5 | 625.4 |
| 12 | 569.4 | 569.3 |
| 13 | 596.4 | 596.5 |
| 14 | 625.5 | 625.4 |
| 15 | 680.5 | 680.5 |
| 16 | 738.5 | 738.4 |
| 17 | 738.5 | 738.6 |
| 18 | 738.5 | 738.6 |


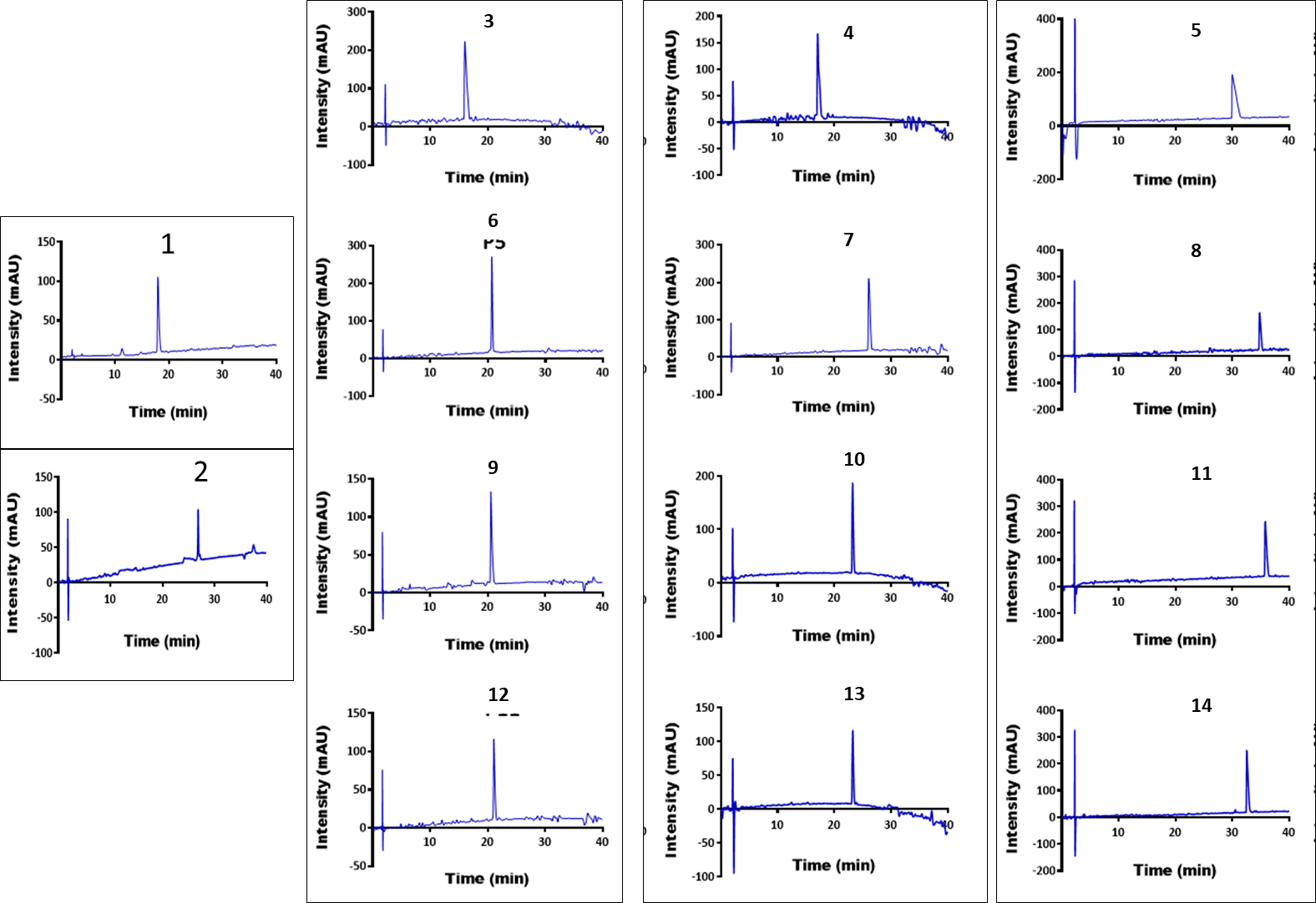


Figure S1: HPLC traces of all peptides synthesised in this study


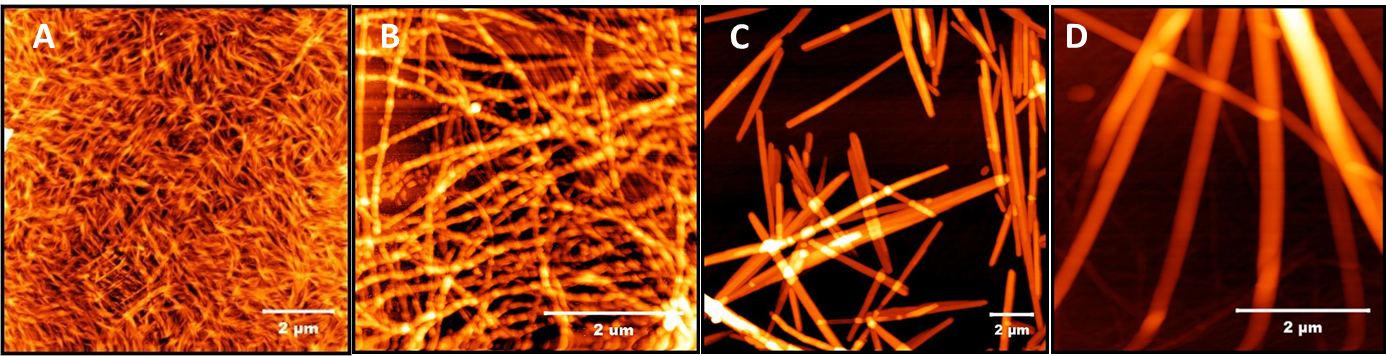


**Figure S2**: AFM images of fibers derived from the self-assembly of peptides **3** (A), **6** (B), **9** (C) and **12** (D).


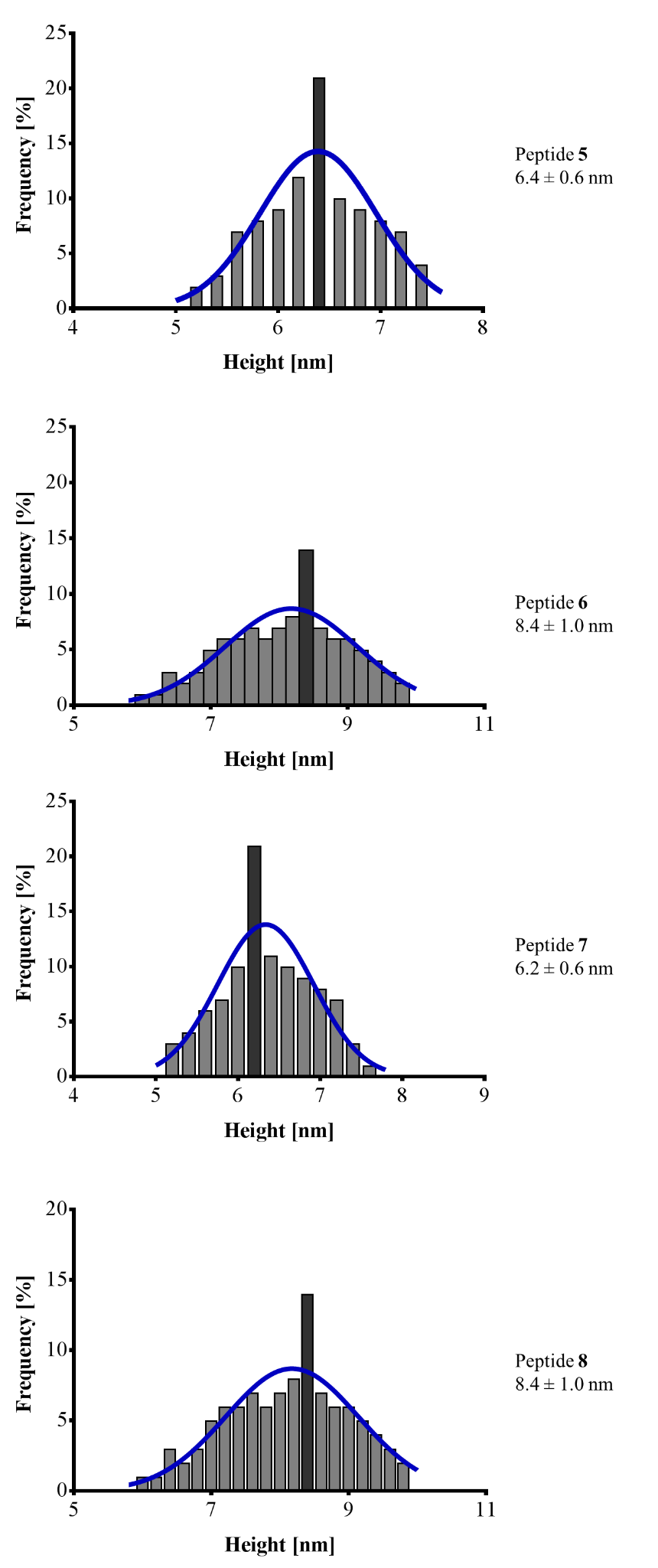


**Figure S3**: The height distribution and averages for peptides **5-8** (C14 and C16 at the N-terminus and residue 1), as measured by AFM.


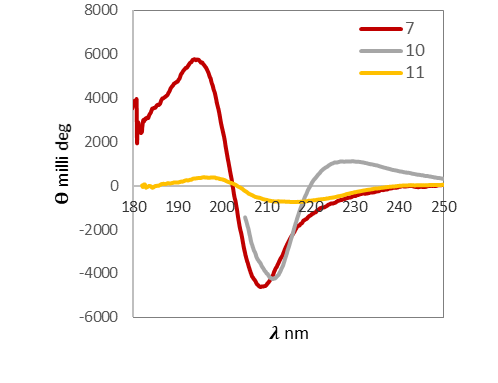


**Figure S4**: CD analysis of lipidated peptides **7, 10 and 11**


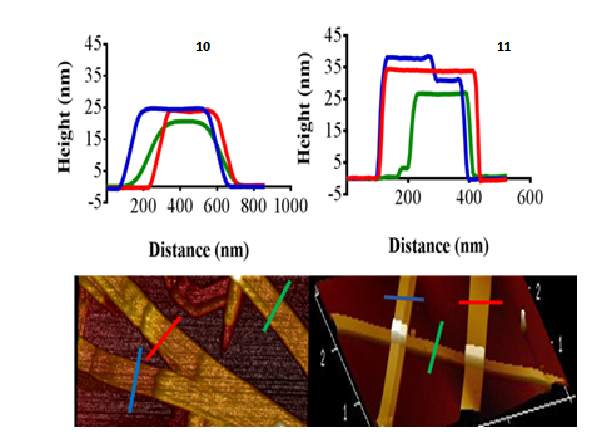


**Figure S5**: Height profiles of three nanobelts for peptides **10** and **11** (C14 and C16 at residue 2) measured by AFM with corresponding AFM images shown below for each profile**.**


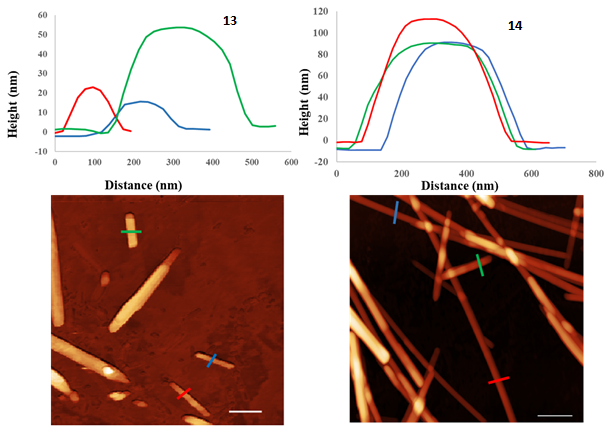


**Figure S6**: The height profiles of three nanobelts for peptides **13** and **14** (C14 and C16 at residue 3) measured by AFM with corresponding AFM images shown below for each profile**.**


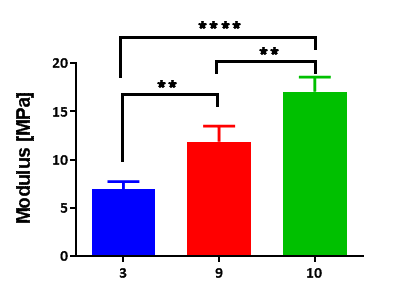


**Figure S7:** Stiffness of nanobelts formed by assembly of lipidated peptides 5 (C12), **10** (C14) and **11** (C16) measured by AFM (PF-QNM) in water. One-way ANOVA Tukey's multiple comparisons test was used for the analysis where *p* values are ˂ 0.01 (**) and ˂ 0.0001 (****), 5 nanobelts per group, mean + SD shown).
